# Case Report: Spontaneous coronary artery dissection with intramural hematoma involving the LCX and the left main segment between the bifurcation and the ostium

**DOI:** 10.3389/fcvm.2026.1926533

**Published:** 2026-09-25

**Authors:** Yulong Liu, Xin Yang, Yang Zhang, Yaling Wang

**Affiliations:** 1 Department of Cardiology, The Second Hospital of Hebei Medical University, Shijiazhuang, Hebei, China; 2 Department of Cardiac Surgery, Shijiazhuang People's Hospital, Shijiazhuang, Hebei, China

**Keywords:** case report, conservative management, intravascular ultrasound, left circumflex coronary artery, left main bifurcation, spontaneous coronary artery dissection, spontaneous coronary intramural hematoma

## Abstract

**Background:**

Spontaneous coronary artery dissection (SCAD) may initially present as acute coronary syndrome and be managed under a presumed ST-segment elevation myocardial infarction pathway before the vessel wall diagnosis is recognized. Management is particularly challenging when extensive intramural hematoma involves left main or bifurcation-related anatomy.

**Case Presentation:**

A 47-year-old woman presented with recurrent chest pain, cardiac biomarker elevation, and inferior/posterior ST-segment abnormalities. Before SCAD was recognized, recurrent inferior ST-segment elevation was treated under a presumed STEMI pathway, including thrombolysis. Angiography showed a distal left circumflex coronary artery (LCX) double-lumen-like appearance with TIMI grade 3 flow. Intravascular ultrasound (IVUS) demonstrated a focal distal LCX intimal disruption/fenestration; extensive intramural hematoma involving the imaged distal, mid, and proximal LCX; the LCX ostium; and the imaged left main segment between the bifurcation and the ostium, with marked true-lumen compression. Given hemodynamic stability and the absence of refractory ischemia, conservative management was selected after balancing the anatomical concern against the procedural risks of revascularization. At approximately 3 months, angiography and IVUS showed disappearance of the distal angiographic abnormality and resolution of intramural hematoma within the reassessed segments. During follow-up through approximately 6 years after discharge, the patient reported no recurrent chest pain or unplanned rehospitalization.

**Conclusion:**

IVUS clarified lesion morphology and extent not fully defined by angiography, illustrating the complementary roles of angiographic and vessel wall information in case-specific risk assessment. Subsequent resolution does not establish treatment efficacy or support general treatment recommendations. The initiating event and direction of hematoma spread could not be determined from the available imaging.

## Introduction

Spontaneous coronary artery dissection is an important non-atherosclerotic cause of acute coronary syndrome, particularly in women with few conventional atherosclerotic risk factors (1–3). The clinical and angiographic presentation can overlap with atherosclerotic acute coronary syndrome, and spontaneous coronary artery dissection (SCAD) may not be recognized at the time of initial ST-segment elevation, biomarker elevation, or recurrent chest pain (1, 2, 4). In practice, the early phase may therefore be managed as presumed STEMI before the vessel wall diagnosis becomes clear. Because SCAD may involve intramural bleeding, the effects of antiplatelet and anticoagulant treatment administered before the vessel wall diagnosis is recognized remain uncertain (1, 2).

Management of SCAD is often conservative when coronary flow is preserved and the patient is clinically stable, because spontaneous healing is common and revascularization can be technically challenging (1, 2, 4, 5). However, this principle is harder to apply when intramural hematoma (IMH) involves left main or bifurcation-related anatomy. Abrupt compromise of the left main or bifurcation-related region could reduce inflow to one or both major left coronary branches, jeopardizing a large myocardial territory. The potential consequences of progression at this location are greater than for distal-vessel disease, supporting particularly close monitoring and timely reassessment (1, 2). Existing guidance favors revascularization when ongoing ischemia, hemodynamic instability, or clear left main flow compromise is present, but it does not provide a simple threshold for a clinically stable patient with preserved coronary flow and extensive left main/bifurcation-related intramural hematoma (1, 2). These cases therefore require individualized integration of anatomy, flow, symptoms, hemodynamic status, and the technical risks of revascularization.

Conservative management and spontaneous healing in clinically stable SCAD with preserved coronary flow are established and are not the contribution of this report. The relevant information gap is that preserved angiographic flow may coexist with vessel wall disease whose anatomical extent and effect on the true lumen are not fully defined by angiography alone. In this patient, angiography showed a distal left circumflex coronary artery (LCX) double-lumen-like appearance with Thrombolysis in Myocardial Infarction (TIMI) grade 3 flow, whereas intravascular ultrasound (IVUS) demonstrated extensive intramural hematoma involving the imaged distal, mid, and proximal LCX; the LCX ostium; and the imaged left main segment between the bifurcation and the ostium, with marked true-lumen compression and a baseline left main minimum lumen area of 8.39 mm^2^. Baseline quantitative IVUS assessment and paired follow-up IVUS on day 111 documented the vessel wall abnormality and its resolution within the reassessed segments. The contribution of this case is therefore an integrated quantitative and longitudinal illustration of the angiography–IVUS information gap, rather than a new treatment strategy.

## Case presentation

Several days before seeking medical attention, a 47-year-old woman with no documented history of hypertension, diabetes, dyslipidemia, smoking, alcohol use, or a family history of coronary artery disease or hereditary disease developed intermittent, predominantly right-sided chest pain associated with nausea; each episode resolved spontaneously within several minutes. Nocturnal chest pain accompanied by nausea and vomiting subsequently prompted evaluation at a local hospital. The overall clinical timeline is summarized in Figure 1.

**Figure 1:**
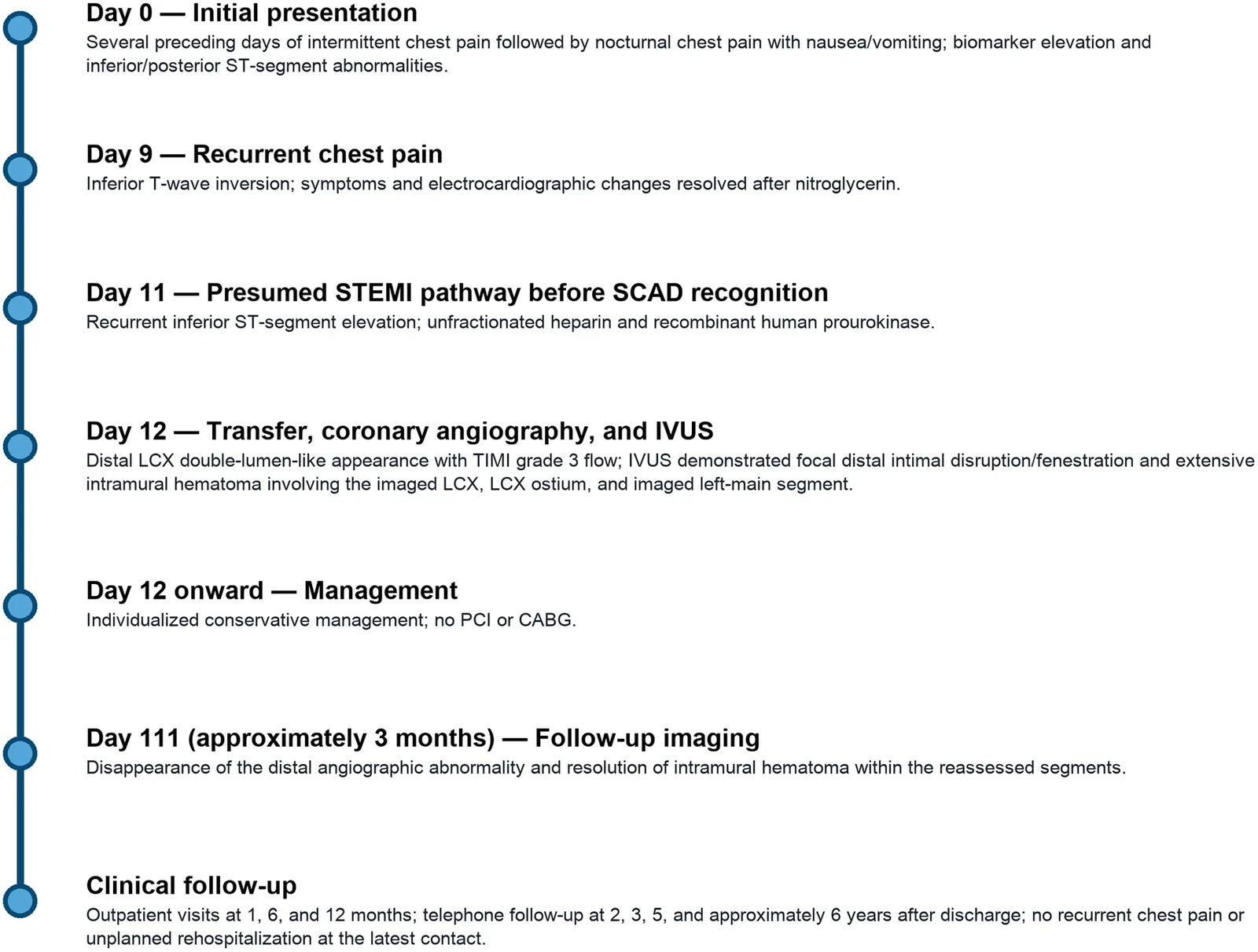
Clinical timeline. Day 0 denotes the initial presentation at the referring hospital. Acute-phase and imaging events are shown in days relative to this reference point; longer-term follow-up is shown in months or years.

At the initial local-hospital presentation, an 18-lead electrocardiogram showed slight ST-segment elevation in leads II, III, aVF, and V7–V9. A repeat electrocardiogram shortly afterward and subsequent serial recordings initially showed no clear dynamic change, and cardiac biomarkers were positive. Before coronary anatomy had been defined, she was treated for presumed acute coronary syndrome. Nine days later, at approximately 09:00, recurrent chest discomfort and chest pain were accompanied by T-wave inversion in leads II, III, and aVF; the symptoms and electrocardiographic changes resolved within approximately 15 min after nitroglycerin therapy. Two days later, at approximately 19:00, recurrent chest pain was accompanied by dynamic ST-segment elevation in leads II, III, and aVF and pathological Q waves in leads III and aVF. She was treated under a presumed STEMI pathway, including thrombolysis, with subsequent symptomatic improvement. Coronary angiography had not been performed at the referring hospital.

The following day, she was transferred to our hospital. On arrival, her blood pressure was 100/70 mmHg, consistent with her usual blood pressure, and oxygen saturation was normal. She was hemodynamically stable without refractory ongoing ischemia. Coronary angiography and IVUS were performed at approximately 11:00.

### Diagnostic assessment

The initial clinical picture was compatible with acute coronary syndrome: recurrent chest pain; slight ST-segment elevation in leads II, III, aVF, and V7–V9; positive cardiac biomarkers: and later recurrent inferior ST-segment elevation with pathological Q waves. Before coronary imaging clarified the diagnosis, these findings led to treatment under presumed acute coronary syndrome and STEMI pathways.

Available cardiac troponin I (cTnI) measurements during the referring-hospital course were as follows. At initial presentation (day 0), cTnI was 1.46 ng/mL (laboratory upper reference limit, 1.00 ng/mL). Subsequent measurements, reported with a different laboratory upper reference limit, were 16.708 ng/mL at 14:20 on day 9 and 11.343 ng/mL at 04:15 on day 10 (upper reference limit, 0.040 ng/mL for both), indicating a decline between those measurements. No cTnI result contemporaneous with the recurrent ST-segment elevation and thrombolysis on day 11 was identified in the records reviewed; therefore, these measurements do not define the complete temporal profile.

After transfer, coronary angiography showed a distal LCX double-lumen-like appearance suggestive of SCAD, with TIMI grade 3 flow. The angiographic appearance did not fully define the underlying lesion morphology, intramural hematoma extent, or true-lumen compression. Representative baseline coronary angiographic images are shown in Figure 2. The full set of available de-identified index coronary angiographic cine runs, including left and right coronary projections, is provided as Supplementary Video 3.

**Figure 2:**
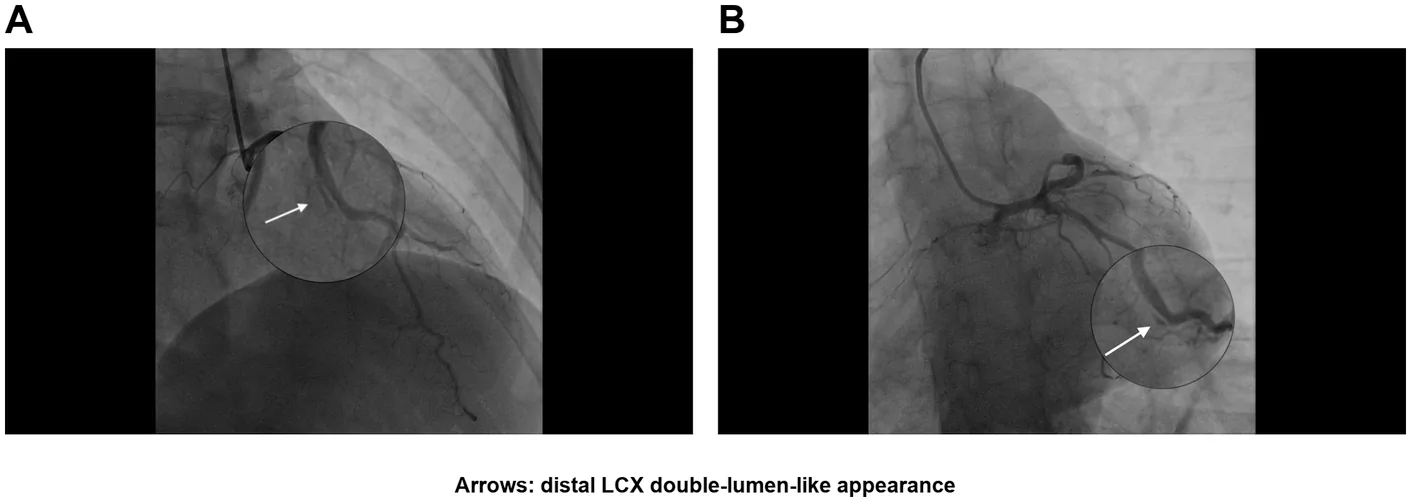
Baseline coronary angiography. Baseline angiography in the right anterior oblique (RAO) cranial **(A)** and left anterior oblique (LAO) caudal (spider) **(B)** projections showed a double-lumen-like appearance in the distal left circumflex coronary artery (LCX) with TIMI grade 3 flow, suggesting coronary dissection. Arrows indicate the distal LCX double-lumen-like angiographic abnormality in both projections. Angiography did not fully define the underlying lesion morphology, intramural hematoma extent, or true-lumen compression subsequently demonstrated by intravascular ultrasound.

At the time of the index procedure, optical coherence tomography (OCT) was not available at our center; IVUS was therefore used to clarify the lesion morphology and extent. IVUS demonstrated a focal distal LCX intimal disruption/fenestration with an intima-media flap, extensive intramural hematoma, and marked true-lumen compression. The pullback sequence defined intramural hematoma within the imaged distal, mid, and proximal LCX; the LCX ostium; and the imaged left main segment between the bifurcation and the ostium. Within the imaged left main segment, IVUS showed intramural hematoma, but no intimal disruption/fenestration or dissection flap was identified. The baseline pullback reached the left main ostial region (frame 5339), marking the most proximal extent of the baseline pullback; the left main minimum lumen area at frame 4684 was 8.39 mm^2^. The left anterior descending (LAD) take-off was visualized during the LCX-to-left-main pullback, but no separate LAD IVUS pullback was performed; therefore, LAD intramural hematoma could neither be confirmed nor excluded by IVUS. The imaging did not establish the initiating site or direction of hematoma spread. Representative baseline IVUS frames are shown in Figure 3, and the complete baseline IVUS pullback is provided as Supplementary Video 1.

**Figure 3:**
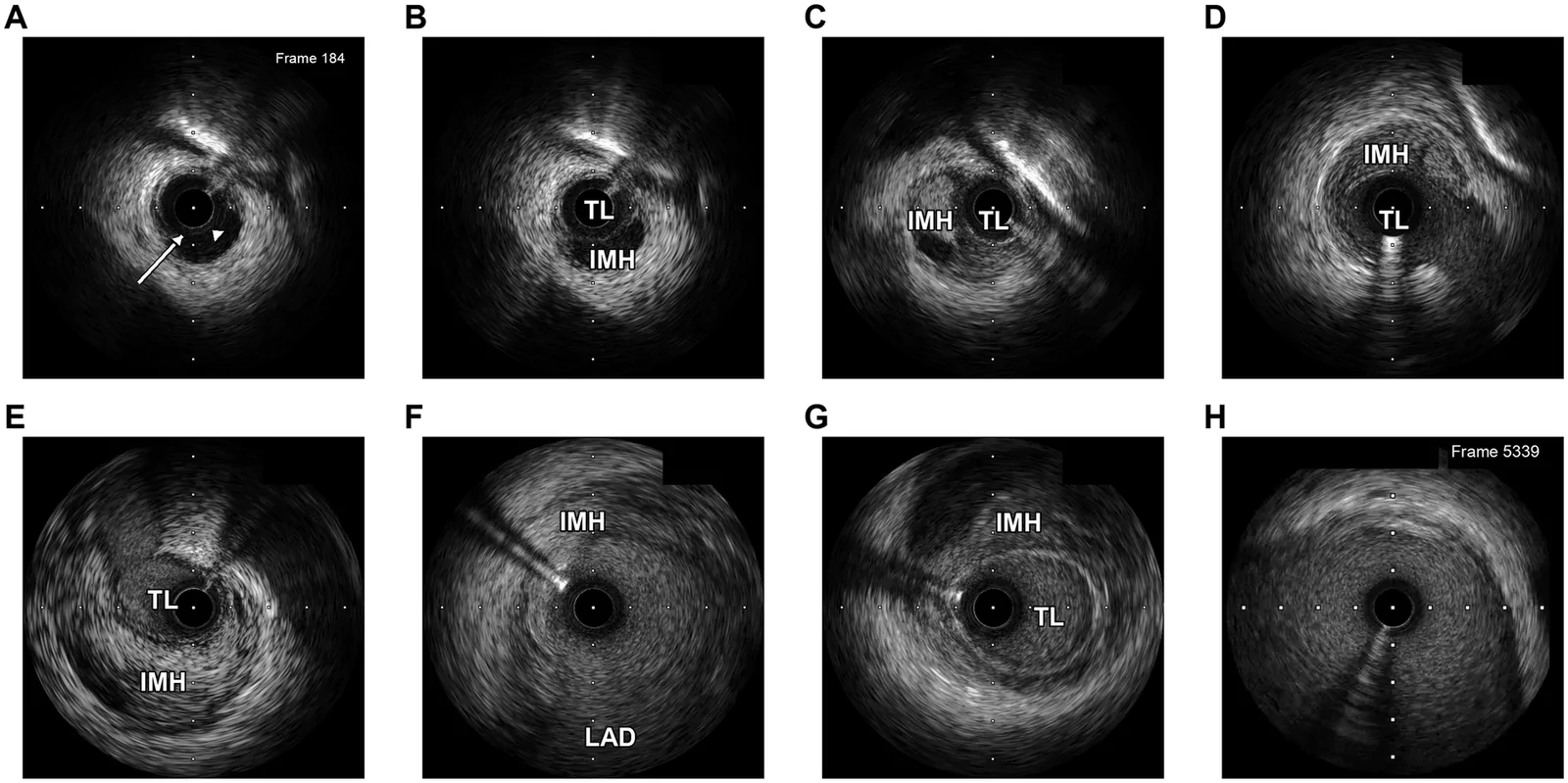
Baseline IVUS demonstrating distal LCX intimal disruption/fenestration and extensive intramural hematoma. **(A)** Focal distal LCX intimal disruption/fenestration with an intima-media flap, frame 184; **(B)** distal LCX, frame 246; **(C)** segment corresponding to the angiographic double-lumen-like/contrast-staining region, frame 1073; **(D)** mid LCX, frame 2126; **(E)** proximal LCX, frame 3391; **(F)** LCX ostium/LAD take-off bifurcation, frame 4447; **(G)** left main shaft at the minimum-lumen-area measurement region, frame 4684 (minimum lumen area, 8.39 mm^2^); and **(H)** left main ostial region, frame 5339, marking the most proximal extent of the baseline pullback. In panel A, the arrow and arrowhead indicate the focal distal LCX intimal disruption/fenestration and intima-media flap, respectively. In panels **B**–**E** and **G**, TL and IMH indicate the true lumen and intramural hematoma, respectively. In panel **F**, LAD labels the visualized LAD take-off and IMH indicates intramural hematoma; no separate LAD IVUS pullback was performed. Serial frames demonstrated intramural hematoma involving the imaged distal, mid, and proximal LCX, the LCX ostium, and the imaged left main segment between the bifurcation and the ostium, with marked true-lumen compression. Within the imaged left main segment, no intimal disruption/fenestration or dissection flap was identified. The images establish morphology and anatomical extent without identifying the initiating site or direction of hematoma spread.

### Therapeutic intervention

Before coronary anatomy was defined at the referring hospital, the patient received loading doses of aspirin 300 mg and clopidogrel 300 mg, followed by aspirin 100 mg once daily and clopidogrel 75 mg once daily. Nadroparin calcium 4,100 anti-Xa IU was administered subcutaneously every 12 h for 7 days and then discontinued. During recurrent ST-segment elevation on day 11, unfractionated heparin was administered intravenously at 60 U/kg (3,720 U for a body weight of 62 kg), followed by recombinant human prourokinase 50 mg (20 mg intravenously over 3 min and then 30 mg by intravenous infusion over 30 min). After thrombolysis, unfractionated heparin was infused at 12 U/kg/h (744 U/h), adjusted to an activated partial thromboplastin time target of 50–70 s, and continued overnight.

The ongoing unfractionated heparin infusion was discontinued before coronary angiography after transfer. During the procedure, an additional 3,000 U of unfractionated heparin was administered based on the activated clotting time. Neither percutaneous coronary intervention (PCI) nor coronary artery bypass grafting (CABG) was performed. Conservative management was selected because TIMI grade 3 flow, hemodynamic stability, and the absence of refractory ongoing ischemia supported observation. The patient had no shock, acute heart failure, malignant arrhythmia, or need for mechanical circulatory support. In contrast, intramural hematoma involving the LCX ostium and the imaged left main segment with marked true-lumen compression created anatomical concern. The anticipated procedural hazards of SCAD revascularization also entered this case-specific balance.

Following diagnostic clarification, the preprocedural unfractionated heparin infusion was not resumed and clopidogrel was discontinued. Aspirin was continued without interruption at 100 mg once daily, and bisoprolol fumarate 2.5 mg once daily was prescribed. Bisoprolol was subsequently discontinued because of low blood pressure, whereas aspirin 100 mg once daily was continued as monotherapy throughout follow-up. This regimen represents the individualized treatment used for this patient and should not be interpreted as evidence that aspirin monotherapy is superior to dual antiplatelet therapy in other patients with non-stented SCAD.

### Follow-up and outcomes

After discharge, the patient attended an outpatient visit at 1 month. On day 111 (approximately 3 months), while clinically stable and asymptomatic, she underwent repeat coronary angiography and IVUS. Angiography showed disappearance of the distal LCX double-lumen-like appearance with TIMI grade 3 flow. IVUS demonstrated resolution of intramural hematoma and restoration of the true lumen within the corresponding reassessed distal, mid, proximal, and ostial LCX segments and the corresponding reassessed left main segment. Representative follow-up angiographic and IVUS images are shown in Figure 4; the follow-up IVUS pullback and the full set of available de-identified follow-up coronary angiographic cine runs are provided as Supplementary Videos 2 and 4, respectively. Subsequent follow-up comprised outpatient visits at 6 months and 1 year, telephone follow-up at 2, 3, and 5 years, and a final telephone contact during manuscript preparation, approximately 6 years after discharge. At the latest contact, the patient reported no recurrent chest pain or unplanned rehospitalization.

**Figure 4:**
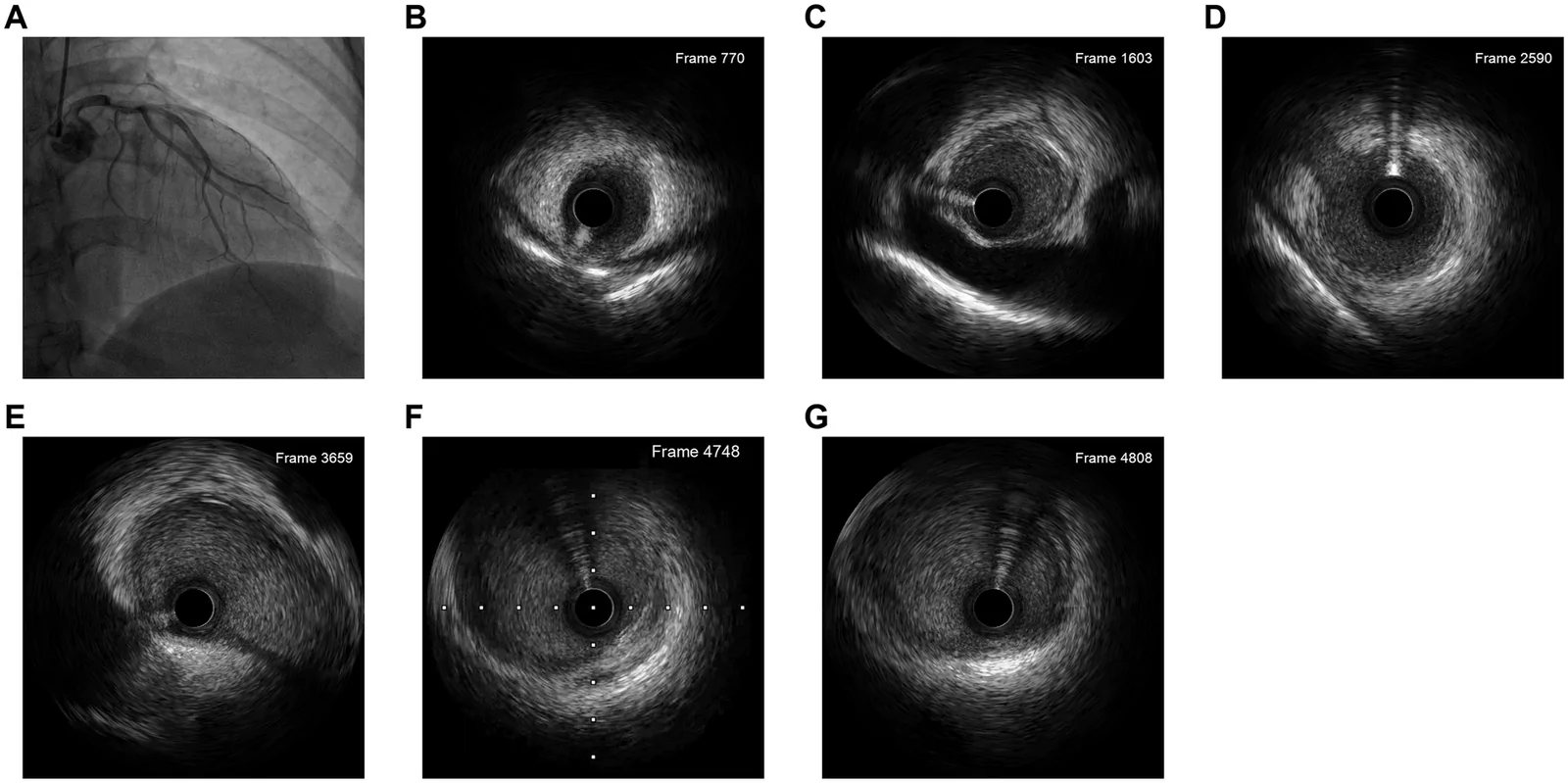
Follow-up angiography and IVUS at approximately 3 months. **(A)** Follow-up coronary angiography in the RAO cranial projection, frame 25, showing disappearance of the prior distal LCX double-lumen-like appearance with TIMI grade 3 flow; **(B)** distal LCX, IVUS frame 770; **(C)** corresponding reassessed LCX segment, IVUS frame 1603; **(D)** mid-LCX, IVUS frame 2590; **(E)** proximal LCX, IVUS frame 3659; **(F)** distal left main bifurcation, IVUS frame 4748; and **(G)** shaft of the left main coronary artery, IVUS frame 4808. Representative IVUS frames show resolution of intramural hematoma and restoration of the true lumen within the reassessed segments.

## Discussion

In this case, angiography showed a distal LCX double-lumen-like appearance but did not fully define lesion morphology, the extent of intramural hematoma, or true-lumen compression. IVUS demonstrated a focal intimal disruption/fenestration with an intima-media flap in the distal LCX. It also showed extensive intramural hematoma involving the imaged distal, mid, and proximal LCX, the LCX ostium, and the imaged left main segment between the bifurcation and the ostium, with marked true-lumen compression. The left main minimum lumen area was 8.39 mm^2^. These findings informed management. Intracoronary imaging may be considered when angiography alone does not establish the diagnosis or adequately characterize lesion extent and when the additional information could alter management, while recognizing the risk of iatrogenic extension (1, 2, 6). OCT offers higher spatial resolution for fine intimal and luminal structures but requires blood clearance, usually with contrast injection, and has more limited tissue penetration; IVUS provides deeper vessel wall penetration without blood clearance but has lower spatial resolution for small intimal disruptions or fenestrations (1, 2).

Conservative management and spontaneous healing in stable SCAD with preserved flow, including left main presentations, have been reported previously, as has serial IVUS documentation of resolution in another coronary distribution (7–9). The contribution of this report instead lies in the integrated record of a clinically relevant discordance: TIMI grade 3 flow and clinical stability on the one hand, and extensive IVUS-defined intramural hematoma with marked true-lumen compression in the imaged LCX, LCX ostium, and imaged left main segment on the other. Baseline quantitative assessment and paired follow-up IVUS on day 111 documented the vessel wall abnormality and its subsequent resolution within the reassessed segments. This record illustrates how intracoronary imaging can refine anatomical risk assessment when angiographic flow appears relatively reassuring; it does not establish a generally applicable treatment strategy or the superiority of IVUS-guided management.

The coexistence of focal distal intimal disruption/fenestration and extensive intramural hematoma raises a mechanistic question. Two principal mechanisms have been proposed for SCAD: an inside-out model, in which primary intimal disruption permits blood to enter the vessel wall, and an outside-in model, in which primary intramural hemorrhage may later produce intimal fenestration (1, 2). Serial angiographic observations have shown that some initially isolated intramural hematomas develop intimal dissection on early repeat imaging (10). In addition, OCT studies have found greater external elastic lamina expansion and a larger false-lumen area in non-fenestrated lesions, supporting an outside-in sequence in at least some cases (11). The disruption and hematoma were each compatible with either mechanism; single-time-point IVUS captured their coexistence rather than their temporal sequence, and neither morphology was mechanism-specific. In the absence of time-sequential acute imaging, OCT, pressure/flow measurements, or pathological evidence, the temporal order of these findings, the function of the disruption, and the direction of hematoma spread could not be determined.

Although the initiating mechanism remained unresolved, management required a separate clinical–anatomical risk assessment. After reviewing the available literature, the treating team balanced IVUS-defined anatomical risk against the clinical and angiographic features described earlier and the hazards of revascularization. Intramural hematoma involving the LCX ostium and the imaged left main segment, with marked true-lumen compression, raised concern about possible compromise of left main or branch perfusion, whereas the absence of refractory ongoing ischemia supported observation. The literature reviewed favored conservative management in stable SCAD with maintained flow (1, 2). The AHA statement identified ongoing ischemia, hemodynamic instability, and left main artery dissection as high-risk situations warranting consideration of urgent PCI or CABG (1), but neither source provided a specific intervention threshold for this anatomical–clinical combination. PCI risked false-lumen wiring, iatrogenic extension, and side-branch compromise; native-vessel healing could limit graft patency after CABG (1, 2). These considerations made the immediate net benefit of revascularization uncertain and shifted the balance toward conservative management.

A separate management issue concerns the antithrombotic and thrombolytic treatment administered before SCAD was recognized. The interhospital course illustrates the difficulty of managing an ACS/STEMI presentation before coronary anatomy is defined. Aspirin, clopidogrel, nadroparin, unfractionated heparin, and thrombolysis preceded recognition of SCAD. Antiplatelet and anticoagulant therapy could theoretically promote intramural bleeding, but causal effects are unproven (1, 2). The DISCO registry reported an observational association between dual antiplatelet therapy and adverse outcomes in conservatively managed SCAD, which does not establish treatment-related hematoma extension or distal intimal disruption (12). No dedicated single antiplatelet therapy (SAPT)-versus-dual antiplatelet therapy (DAPT) comparison exists for left main involvement or extensive intramural hematoma; this case does not establish SAPT superiority or aspirin-mediated healing. Historical reports described dissection or hematoma extension after thrombolysis (13, 14), whereas a later observational study found no statistically significant outcome difference in SCAD-STEMI with versus without thrombolysis, although paired intracoronary imaging was unavailable (15). This case supports early consideration of SCAD and reassessment of antithrombotic treatment once imaging establishes the diagnosis (1, 2).

The decision to perform invasive reassessment on day 111 was individualized. Persistence of the baseline vessel wall abnormality in the anatomically high-risk LCX ostium and imaged left main segment could have influenced continued management. Angiography reassessed the lumen, whereas IVUS evaluated the abnormality that dominated the baseline examination. Although the contemporaneous record lacked a separate statement of indication, this explanation relies only on baseline information rather than the later favorable result. Observational data make approximately 3 months a plausible reassessment interval rather than a fixed recommendation; CCTA is non-invasive but may be limited in distal or small-caliber vessels (1, 2, 5, 16).

These observations should be interpreted in light of several limitations. Acute-phase imaging consisted of a single IVUS examination, precluding assessment of early hematoma progression and determination of whether intimal disruption or intramural hemorrhage occurred first. IVUS evaluation was confined to the LCX and left main segments included in the pullback. Because no separate LAD pullback was performed, LAD intramural hematoma could neither be confirmed nor excluded by IVUS. Coronary imaging was not performed before antithrombotic and thrombolytic treatment, so the contribution of these therapies to hematoma evolution could not be determined. The management decision and imaging outcome from this single case should not be generalized or used to support routine invasive surveillance or repeat IVUS.

## Patient perspective

The patient described the most difficult part of the illness as the uncertainty of not knowing what was happening to her body, especially when she experienced recurrent pain at the local hospital. After the diagnosis of spontaneous coronary artery dissection was explained and conservative management without stent implantation or open-heart surgery was recommended, she initially felt uncertain but decided to trust the clinical judgment of her doctors. After follow-up, she reported feeling physically similar to her pre-illness state but noted that the experience made her more mindful of her health and less likely to take it for granted.

## Conclusion

In this patient, TIMI grade 3 flow coexisted with extensive IVUS-defined intramural hematoma involving the LCX, its ostium, and the imaged left main segment, with marked true-lumen compression. Conservative management was selected after a case-specific balance of clinical stability, anatomical concern, and the anticipated hazards of revascularization. Follow-up angiography and IVUS on day 111 documented resolution within the reassessed segments. This observation is confined to a single case, and its temporal association with conservative management does not establish treatment efficacy or support general treatment recommendations. IVUS provided vessel wall information not fully defined by angiography, while the initiating event and direction of hematoma spread could not be determined from the available imaging.

## Data Availability

The original contributions presented in the study are included in the article/Supplementary Material; further inquiries can be directed to the corresponding authors.
