# Supplementary material for: Case Report: Spontaneous coronary artery dissection with intramural hematoma involving the LCX and the left main segment between the bifurcation and the ostium
Source: Front Cardiovasc Med. 2026 Sep 25;13:1926533. doi: 10.3389/fcvm.2026.1926533 (PMC13649403; doi:10.3389/fcvm.2026.1926533)
Supplement: Supplementary file 1 [file Datasheet1.docx]

**CARE Checklist**

Completed against the revised manuscript render dated 26 August 2026

| **Item** | **Topic** | **CARE requirement** | **Manuscript location** |
| --- | --- | --- | --- |
| 1 | Title | The diagnosis or intervention of primary focus followed by the words 'case report'. | p. 1, lines 1–3 |
| 2 | Keywords | 2-5 key words identifying diagnoses or interventions, including 'case report'. | p. 2, lines 9–12; 7 keywords are used, within Frontiers' requirement of 5–8 keywords |
| 3a | Abstract | Introduction: what is unique about the case and what it adds to the literature. | p. 1, lines 16–19; p. 2, lines 4–8 |
| 3b | Abstract | The patient's main concerns and important clinical findings. | p. 1, lines 21–30 |
| 3c | Abstract | The primary diagnoses, interventions, and outcomes. | p. 1, lines 21–32; p. 2, lines 1–2 |
| 3d | Abstract | Conclusion: the principal take-away observations from the case. | p. 2, lines 4–8 |
| 4 | Introduction | A brief summary of why the case is notable, with relevant literature. | p. 2, lines 13–36; p. 3, lines 1–9 |
| 5a | Patient information | De-identified patient-specific information. | p. 3, lines 11–16 |
| 5b | Patient information | Primary concerns and symptoms. | p. 3, lines 11–28 |
| 5c | Patient information | Relevant medical, family, and psychosocial history, including genetic information when applicable. | p. 3, lines 11–13; only source-confirmed relevant medical, family, and risk-factor information is reported |
| 5d | Patient information | Relevant past interventions and their outcomes. | p. 3, lines 17–28; Therapeutic Intervention, p. 4, lines 33–37 and p. 5, lines 1–20 |
| 6 | Clinical findings | Relevant physical examination and other significant clinical findings. | p. 3, lines 29–32 |
| 7 | Timeline | Important historical and current information organized as a timeline. | Figure 1, p. 9, lines 2–4 |
| 8a | Diagnostic assessment | Diagnostic methods, including examination, laboratory testing, imaging, and surveys. | Diagnostic Assessment, p. 3, lines 33–36 and p. 4, lines 1–31 |
| 8b | Diagnostic assessment | Diagnostic challenges, including access, financial, cultural, or other barriers when relevant. | Diagnostic uncertainty and imaging limitations: p. 4, lines 11–31; Discussion, pp. 5–8 |
| 8c | Diagnostic assessment | Diagnosis, including differential diagnoses considered. | Diagnostic Assessment, p. 4, lines 11–31 |
| 8d | Diagnostic assessment | Prognostic characteristics when applicable. | Flow, anatomical concern, and clinical stability: p. 4, lines 11–31 and p. 5, lines 5–13 |
| 9a | Therapeutic intervention | Types of intervention, including pharmacologic, surgical, preventive, and self-care measures. | Therapeutic Intervention, p. 4, lines 33–37 and p. 5, lines 1–20 |
| 9b | Therapeutic intervention | Administration of the intervention, including dose, strength, and duration when available. | Therapeutic Intervention, p. 4, lines 33–37 and p. 5, lines 1–20 |
| 9c | Therapeutic intervention | Changes in intervention, with explanations. | Therapeutic Intervention, p. 5, lines 5–20 |
| 9d | Therapeutic intervention | Rationale for the selected intervention. | Therapeutic Intervention, p. 5, lines 8–13; Discussion, pp. 6–7 |
| 10a | Follow-up and outcomes | Clinician- and patient-assessed outcomes. | Follow-Up and Outcomes, p. 5, lines 21–33; Patient Perspective, p. 8, lines 3–10 |
| 10b | Follow-up and outcomes | Important follow-up diagnostic and other test results. | Follow-Up and Outcomes, p. 5, lines 22–30; Figure 4, p. 12, lines 2–9 |
| 10c | Follow-up and outcomes | Intervention adherence and tolerability. | Therapeutic Intervention, p. 5, lines 14–20; Follow-Up and Outcomes, p. 5, lines 21–33 |
| 10d | Follow-up and outcomes | Adverse and unanticipated events. | Follow-Up and Outcomes, p. 5, lines 21–33; outcomes are limited to documented and patient-reported events |
| 11a | Discussion | Strengths and limitations associated with the case report. | Discussion, p. 5, line 34 through p. 8, line 2, including the consolidated limitations paragraph |
| 11b | Discussion | Relevant medical literature. | Discussion, pp. 5–8; References, pp. 13–15 |
| 11c | Discussion | Scientific rationale for conclusions, including assessment of possible cause and effect. | Mechanistic and causal boundaries: p. 6, lines 21–33; management reasoning: p. 6, lines 34–39 and p. 7, lines 1–8; limitations: p. 7, lines 32–38 and p. 8, lines 1–2 |
| 11d | Discussion | Primary take-away lessons in a one-paragraph conclusion. | Conclusion, p. 8, lines 11–21 |
| 12 | Patient perspective | The patient shares her perspective or experience when appropriate. | Patient Perspective, p. 8, lines 3–10 |
| 13 | Informed consent | The patient provided informed consent for publication. | Consent for Publication, p. 12, lines 20–22 |

Note: Page and line references correspond to the revised manuscript render. Where a CARE element is not separately applicable or not independently assessed, this is stated explicitly rather than inferred.
