# Supplementary material for: Case Report: Spontaneous coronary artery dissection with intramural hematoma involving the LCX and the left main segment between the bifurcation and the ostium
Source: Front Cardiovasc Med. 2026 Sep 25;13:1926533. doi: 10.3389/fcvm.2026.1926533 (PMC13649403; doi:10.3389/fcvm.2026.1926533)
Supplement: Supplementary file 2 [file Datasheet2.docx]

**Supplementary Video Legends**

Manuscript ID: 1926533

# Supplementary Video 1

Supplementary Video 1. Baseline IVUS pullback. The de-identified baseline IVUS pullback demonstrates focal distal LCX intimal disruption/fenestration with an intima-media flap, extensive intramural hematoma involving the imaged distal, mid, and proximal LCX, the LCX ostium, and the imaged left-main segment between the bifurcation and the ostium, with marked true-lumen compression. Within the imaged left-main segment, intramural hematoma was present, but no intimal disruption/fenestration or dissection flap was identified. The LAD take-off was visualized during the LCX-to-left-main pullback, but no separate LAD IVUS pullback was performed; therefore, LAD intramural hematoma could neither be confirmed nor excluded by IVUS. The pullback reaches the left-main ostial region at frame 5339. The sequence defines the imaged anatomical extent without establishing the initiating site or direction of hematoma spread.

# Supplementary Video 2

Supplementary Video 2. Follow-up IVUS pullback at approximately 3 months. The de-identified follow-up IVUS pullback demonstrates resolution of intramural hematoma and restoration of the true lumen within the corresponding reassessed distal, mid, proximal, and ostial LCX segments and the corresponding reassessed left-main segment. The terminal landmarks include the distal left main bifurcation at frame 4748 and the shaft of the left main coronary artery at frame 4808.

# Supplementary Video 3

Supplementary Video 3. Baseline coronary angiography. The de-identified video presents the full set of available index coronary angiographic cine runs in the following order: (1) left coronary artery in the left anterior oblique (LAO) caudal (spider) projection; (2) left coronary artery in the right anterior oblique (RAO) caudal projection; (3) left coronary artery in the RAO cranial projection; (4) left coronary artery in the LAO cranial projection; (5) right coronary artery, projection 1; and (6) right coronary artery, projection 2. The distal left circumflex coronary artery (LCX) double-lumen-like appearance with TIMI grade 3 flow is demonstrated in the baseline left-coronary cine runs; the RAO cranial and LAO caudal projections correspond to Figure 2A and Figure 2B, respectively.

# Supplementary Video 4

Supplementary Video 4. Follow-up coronary angiography at approximately 3 months. The de-identified video presents the full set of available follow-up coronary angiographic cine runs in the same order as Supplementary Video 3: (1) left coronary artery in the LAO caudal (spider) projection; (2) left coronary artery in the RAO caudal projection; (3) left coronary artery in the RAO cranial projection; (4) left coronary artery in the LAO cranial projection; (5) right coronary artery, projection 1; and (6) right coronary artery, projection 2. The follow-up left-coronary cine runs demonstrate disappearance of the prior distal LCX double-lumen-like appearance with TIMI grade 3 flow. The RAO cranial projection corresponds to Figure 4A.
